# *What’sPrEP?*: peer navigator acceptability among minority MSM in Washington

**DOI:** 10.1186/s12889-020-8325-5

**Published:** 2020-02-18

**Authors:** Jade Pagkas-Bather, Jahn Jaramillo, Jsani Henry, Vanessa Grandberry, Luis F. Ramirez, Lorenzo Cervantes, Joanne D. Stekler, Michele P. Andrasik, Susan M. Graham

**Affiliations:** 1 Department of Medicine, University of Washington, Seattle, WA USA; 2 Department of Medicine, Section of Infectious Diseases & Global Health, The University of Chicago, 5841 South Maryland Avenue, Chicago, IL 60637 USA; 3 Centers for Disease Control and Prevention, Guatemala City, Guatemala; 4 Seattle & King County Public Health HIV/STD Program, Seattle, WA USA; 5 Center for MultiCultural Health, Seattle, WA USA; 6 Entre Hermanos, Seattle, WA USA; 7 Pierce County AIDS Foundation, Tacoma, WA USA; 8 Fred Hutchinson Vaccine and Infectious Disease Division, Seattle, USA

**Keywords:** HIV prevention, Pre-exposure prophylaxis, Black, Latinx, Men who have sex with men

## Abstract

**Background:**

Peer navigation is a promising strategy to link at-risk minority men who have sex with men (MSM) to HIV prevention services including pre-exposure prophylaxis (PrEP).

**Methods:**

Thirty-two Black and 63 Latinx HIV-negative MSM living in western Washington completed a survey examining attitudes towards peer navigation and PrEP. Factor analysis derived a score for peer navigator acceptability, and linear regression identified associations with this outcome**.**

**Results:**

Forty-eight percent were interested in peer navigation. Being insured, higher sexual stigma, and higher PHQ-9 score were associated with higher acceptability, while higher income and having a regular medical provider were associated with lower acceptability. In multivariable analysis, higher sexual stigma predicted higher acceptability, while higher income predicted lower acceptability. Men preferred that peers be matched on sexual orientation, race, age and culture.

**Conclusion:**

Peer navigation interventions to reach minority men should address stigma, focus on lower-income men, and try to match peers to clients to the extent possible.

## Background

Pre-exposure prophylaxis (PrEP) using oral emtricitabine and tenofovir in a combination pill reduces the risk of HIV transmission if adherence is high or intermittent dosing is timed to sexual activity [1, 2]. In the United States, PrEP has become popular among many White men who have sex with men (MSM), but has, to date, failed to gain a firm foothold among Black and Latinx populations, who are disproportionately impacted by HIV [3]. In 2016, 73% of PrEP users were White, 10% were Black (12% in 2015), and 13% were Latinx [4, 5], similar to the make-up of the general U.S. population [6, 7]. In contrast, White MSM made up only 22% of new HIV infections, while Black MSM made up 31% and Latinx MSM made up 22% of 33,210 new infections in the United States in 2016 [6, 7]. Black and Latinx MSM are disproportionately at risk for HIV compared to White MSM, yet less likely to use PrEP for HIV prevention.

For a number of reasons, Black and Latinx MSM lack equal access to HIV prevention resources. Minority MSM experience discrimination from within and outside their communities, as a result of both race/ethnicity and sexual minority status [8, 9]. Structural and economic inequities, such as lack of health insurance, lower income, higher rates of unemployment, and incarceration further widen the gap between HIV-related health outcomes in Black and Latinx MSM compared to their White counterparts [10–14].

Peer navigators represent one potential strategy to address disparities in HIV acquisition and PrEP uptake among minority MSM, by offering a layperson’s perspective on HIV prevention and helping men link to and navigate the healthcare system. Such assistance may be especially important, given the role of medical mistrust as a barrier to care in the Black and Latinx communities [15, 16]. PrEP-utilizing peer navigators may help combat medical mistrust and fill in PrEP-related knowledge gaps that exist among minority MSM [17–20]. For example, in a cohort of young Black MSM in Chicago, discussions with confidants regarding HIV prevention were associated with greater PrEP awareness (aOR = 2.26, 95% CI 1.00 to 5.09) [21]. Peer navigator interventions targeting the HIV care cascade demonstrate that peers can improve patient engagement and increase medication adherence [22].

In western Washington, Latinx individuals represent a growing population, with many new immigrants from Latin and South America, as well as Latinx Americans migrating to the area [23]. Resettlement often encourages individuals to find new communities with similar culture and language. This process of migration might also encourage gathering and seeking out community, which could facilitate peer navigation among Latinx MSM. Furthermore, the stresses of immigration and the threat of deportation within Latinx communities might increase the importance of peer navigator outreach, as Latinx individuals could be afraid to engage with medical providers, especially those who are not Latinx [24].

In contrast, Black communities in western Washington are dispersing, as many Black families have moved out of metro King County/Seattle due to increased housing costs and gentrification [25, 26]. This migration to the suburbs could make it more difficult for Black MSM to connect to peer navigators, as few LGBTQ organizations are located outside of King County, which includes downtown Seattle [25, 26]. While both Black and Latinx MSM are key populations at higher risk for HIV, Black MSM may be at even higher risk for HIV transmission given the potential for greater social and cultural isolation in western Washington. For example, Black populations are smaller than Latinx populations in King (6.8% vs 9.7%), Pierce (7.5% vs 10.9%), Snohomish (3.5% vs 10.2%), and Thurston (3.4% vs 9.0%) counties, where this study took place [7]. In a study of Chicago residents conducted by the University of Chicago, Black respondents were less likely to identify as lesbian, gay, bisexual, transgender, or queer than Latinx respondents (22% of Latinx respondents vs 14% of Black respondents identified as LGBTQ) [27]. If this is true of western Washington, Black individuals might not see themselves reflected in local LGBTQ organizations despite their outreach efforts, and may therefore be less likely to seek support from them.

In 2017, the Washington State Department of Health funded the Persons at High Risk (PAHR) Navigation Services program funding community-based organizations and agencies in the state to increase PrEP access and HIV testing, as well as healthcare engagement for individuals not being reached using previous strategies [28]. The *What’s PrEP?* study aimed to evaluate the acceptability of peer navigation for PrEP use among Black and Latinx men living in Western Washington, and to identify factors associated with higher or lower acceptability of this approach. In addition, the study aimed to identify which peer navigator characteristics were most important to minority MSM. By identifying modifiable factors associated with acceptability, study findings could be used to identify potential challenges peer navigators might face and to inform the design of programs for effective peer navigation.

## Methods

### Study population

*What’s PrEP?* and its Spanish language version, *Que es PrEP?* was a cross-sectional study that surveyed non-Latinx Black and Latinx cis and transgender MSM. Participants were required to be HIV-negative, male-identified Black or Latinx, English or Spanish-speaking, age 16 or older, sexually active with a male in the past 12 months, and residing in the Snohomish, Thurston, Pierce, or King counties of western Washington. Participants were recruited by flyer distribution at events, Facebook posts, and word of mouth, as well as through local HIV prevention and STD clinics and from local community-based organizations including Entre Hermanos, the Center for Multicultural Health (CMCH), PCAF (Pierce County AIDS Foundation), Project Neon, and Gay City Health Project.

### Procedures and data collection

Participants took a survey online using REDCap or in-person using a printed, self-administered questionnaire (Additional file 1). The survey, which we estimated to take 30–45 min on a computer and up to 60 min on a cell phone, collected information on demographics, HIV risk behaviors, alcohol and drug use, depressive symptoms, sexual stigma, healthcare access, PrEP use and delivery preferences, and interest in a peer navigator for the use of PrEP. Gender identity was characterized using a two-step process to assess sex at birth and current gender identity [29]. Participants were reimbursed promptly using a hard-copy Visa card or electronic Tango card worth $40. A subset of participants underwent an in-depth interview; results of these interviews are reported elsewhere. Names were collected only in order to provide reimbursement or to invite participants for interviews, and surveys were de-identified.

### Measures

#### Peer navigator acceptability

Participants were asked four questions about their willingness to use a peer for appointment reminders, pill-taking reminders, advice about PrEP (e.g., managing side effects, adherence, planning for refills), and discussing privacy or other concerns surrounding PrEP use. Responses were rated as 1 = very harmful/interfering, 2 = somewhat useless or harmful/interfering, 3 = neither useful nor useless, 4 = somewhat useful, or 5 = very useful.

#### Peer navigator attributes

Participants were asked seven questions about how important different attributes of a potential peer were, in terms of matching with respect to race, sexual orientation, age, relationship status, income, culture, and neighborhood. Responses were rated as rated as 1 = not important, 2 = slightly important, 3 = fairly important, 4 = important, or 5 = very important.

#### Sociodemographic factors

Data were collected on participant age, years of education, and monthly income. Race/ethnicity was defined as non-Latinx Black vs Latinx, with the small number of Black Latinx individuals classified as Latinx. Sexual orientation was defined as gay, straight, bisexual, queer, or other. Gender identity was assigned as cisgender if participants were male at birth and identified as male or non-binary/genderqueer and transgender if participants were female at birth and identified as male.

#### Mental health

Sexual stigma was measured using Logie’s modified China MSM Stigma Scale, which evaluates perceived stigma and discrimination faced by MSM as pertains to safety, family, and relationships [30]. Depressive symptoms were measured using the Patient Health Questionnaire (PHQ-9), a self-administered set of questions used to identify depressive symptoms in clinical settings [31]. Disordered alcohol use was measured using the Alcohol Use Disorders Identification Test (AUDIT) [32]. Non-alcohol substance use was measured using the Drug Abuse Screening Test 10 (DAST-10) [33].

#### Health care factors

A series of questions assessed whether participants had health insurance, saw a regular medical provider, had disclosed their MSM status to their provider, had ever tested for HIV, and the time since their last HIV test, and clinic visit [34].

#### Sexual behavior and PrEP use

A series of questions adapted from the 2014 CDC PrEP clinical practice guidelines [35] were asked, to screen for high-risk behaviors in the past 12 months. These included condomless anal sex with an HIV-negative man, ongoing relationship with an HIV-positive male partner, treatment for an STI, use of post-exposure prophylaxis (PEP), use of crystal meth, use of poppers, non-prescription injection drug use, and exchange sex for drugs, money or housing. Additional questions asked about participants’ PrEP awareness and use, as well as whether participants were interested in starting or continuing PrEP.

### Statistical analysis

Descriptive statistics were used to characterize the study population overall and separately for non-Latinx Black and Latinx participants. Chi square or Fisher exact tests were used to examine differences between categorical variables, and Wilcoxon rank sum tests were used to examine differences in continuous variables across categories, including race/ethnicity. Confirmatory factor analysis was conducted to confirm that observed responses to questions regarding participants’ interest in peer navigation for PrEP underlay an unobserved latent variable for peer navigator acceptability. Results of the factor analyses were used to predict an acceptability score (described in results), which was the primary outcome.

Unadjusted linear regression was used to evaluate the association of race/ethnicity, sexual orientation, and other variables of interest with peer navigator acceptability. A full multivariable model was then constructed, including race/ethnicity and sexual orientation a priori and other potential correlates associated with peer navigator acceptability at *p* < 0.10 in unadjusted analysis. A final, limited analysis included the two a priori predictors and variables associated with peer navigator acceptability at p < 0.10 in the full multivariable analysis. A Wald test was conducted to obtain overall *p*-values for categorical variables included in multivariable analysis.

Descriptive statistics were used to report the proportion of participants who rated each peer attribute as “important” or “very important,” both overall and by race/ethnicity category. Participants’ ratings of the importance of each peer navigator attribute were graphed using a stacked horizontal bar chart, with color coding for each Likert scale response. Responses of non-Latinx Black and Latinx participants were compared using Wilcoxon rank sum tests and presented in a bar graph. The correlations between peer navigator attribute ratings and acceptability were examined using Spearman’s correlation coefficient.

### Ethical considerations

Study procedures and survey questions were developed in collaboration with community partners at Entre Hermanos, the Center for Multicultural Health, and the King County Health Department. Feedback from these and other partners including Gay City, was used to improve recruitment materials and survey wording. The study protocol was approved by the University of Washington Human Subjects Division. All participants provided written or electronic informed consent. All data was securely stored on a password protected computer, and all paper forms were stored on a locked unit in a locked cabinet. Data was accessed only by the study investigators.

## Results

Overall, 301 REDCap survey attempts were made, of which 137 were not complete, due to ineligibility or failure to continue to the end, and 164 were complete. Of the 164 completed surveys, 95 were confirmed via e-mail, phone, or in person confirmation of a unique participant. All ninety-five participants were cisgender men; no transgender men enrolled despite efforts to recruit in this category. Thirty-two (34%) of participants were Black and 63 (66%) were Latinx (4 of the Latinx participants were also Black, Table 1). Overall 69 (73%) participants identified as gay, 19 (20%) identified as bisexual, and 5 (5%) identified as queer/other. The median age was 30 (IQR 26–40) years, with a range of 18 to 66 years of age.

**Table 1:** Study population, with comparison of Black and Latinx MSM

Variable	non-Latinx Black *n* = 32 (N, % or median, IQR)	Latinx *n* = 63 (N, % or median, IQR)	*P*-value
Sociodemographic factors			
Sexual orientation			< .001
Gay	15 (47)	54 (86)	
Straight	2 (6)	0 (0)	
Bisexual	13 (41)	6 (9)	
Queer	2 (6)	3 (5)	
Age (years)	42.5 (30–54.5)	29 (25–34)	<.001
Education			0.06
< high school	4 (13)	3 (5)	
high school	8 (25)	7 (11)	
some college	15 (47)	26 (42)	
college	3 (9)	19 (31)	
graduate school	2 (6)	7 (11)	
Insured (yes)	29 (91)	42 (67)	0.01
Income			0.32
$0–$1500	18 (56)	28 (45)	
$1501–$3500	6 (19)	21 (33)	
> $3500	8 (25)	14 (22)	
Foreign Born (yes)	2 (6)	30 (48)	< 0.001
Current PrEP use and interest			
Currently taking PrEP (yes)	8 (25)	25 (40)	
Interested but not taking PrEP	8 (25)	18 (28)	0.19
Not interested in taking PrEP	16 (50)	20 (32)	
Ever prescribed PrEP (yes)	8 (23)	27 (77)	0.32
Ever taken PrEP (yes)	8 (21)	31 (79)	0.02
Mental health			
Stigma score	8 (3–12)	11 (8–14)	0.004
PHQ-9 score	7 (3–10)	7.5 (4–12.5)	0.16
DAST score	1 (0–6)	1 (0–2)	0.13
AUDIT score	5 (3–15)	5.5 (4–11)	0.98
Healthcare factors			
Regular medical provider (yes)	21 (66)	38 (60)	0.30
Disclosed sexual orientation to provider (yes)	13 (41)	34 (54)	0.01
Sexual health			
Ever tested for HIV	29 (91)	58 (92)	0.81
Time since last HIV test^a^			
< 3 months	16 (55)	35 (60)	0.82
3–6 months	5 (17)	12 (21)	
6–12 months	4 (14)	5 (9)	
> 12 months	4 (14)	6 (10)	
Time since last clinic visit (months)	3 (1–5)	2 (1–3)	0.30
High-risk behavior			
Condomless anal sex with HIV negative man	15 (47)	50 (79)	0.001
Relationship with HIV positive partner	1 (3)	7 (11)	0.19
STI treatment in last 12 months	7 (22)	36 (57)	0.002
Use of PEP in last 12 months	2 (6)	16 (25)	0.02
Use of crystal meth	5 (16)	5 (8)	0.25
Use of poppers	8 (25)	27 (43)	0.09
Non-prescription IVDU	1 (3)	3 (5)	0.72
Exchange sex for drugs, money, housing	4 (13)	3 (5)	0.17
Any report of these high-risk behaviors	22 (69)	57 (90)	0.01
Importance of peer attributes			
Peer attributes ranked as			
important/very important			
Same sexual orientation	23 (72)	47 (74)	0.56
Same race	16 (50)	34 (54)	0.07
Same age	16 (50)	40 (63)	0.33
Same culture	13 (41)	28 (44)	0.35
Same neighborhood	9 (28)	27 (43)	0.17
Same income	7 (22)	21 (33)	0.14

^a^ 8 participants did not report the time since their last HIV test

### Differences by race/ethnicity

Black participants were more likely to identify as bisexual or straight rather than gay, relative to Latinx participants (Table 1, *p* < 0.001). In addition, Black participants were older (median 42.5 years vs 29 years, *p* = 0.001) and more likely to be insured (91% vs 67%, *p* = 0.01). Latinx participants were more likely to be foreign born than Black participants (48% vs. 6%, p < 0.001). More Latinx participants were currently using PrEP compared to Black participants (40% vs. 25%, *p* = 0.19), and Latinx participants were more like to have used post-exposure prophylaxis than Black participants (25% vs. 6%, *p* = 0.02). Latinx participants had higher median sexual stigma scores than Black participants (11 vs. 8, *p* = 0.004), although more Latinx participants had disclosed their sexual orientation to their medical provider (54% vs. 41%, *p* = 0.01). Overall, Latinx participants reported more high-risk behaviors than Black participants (details in Table 1).

### PrEP interest and sexual risk

**Fig. 1:**
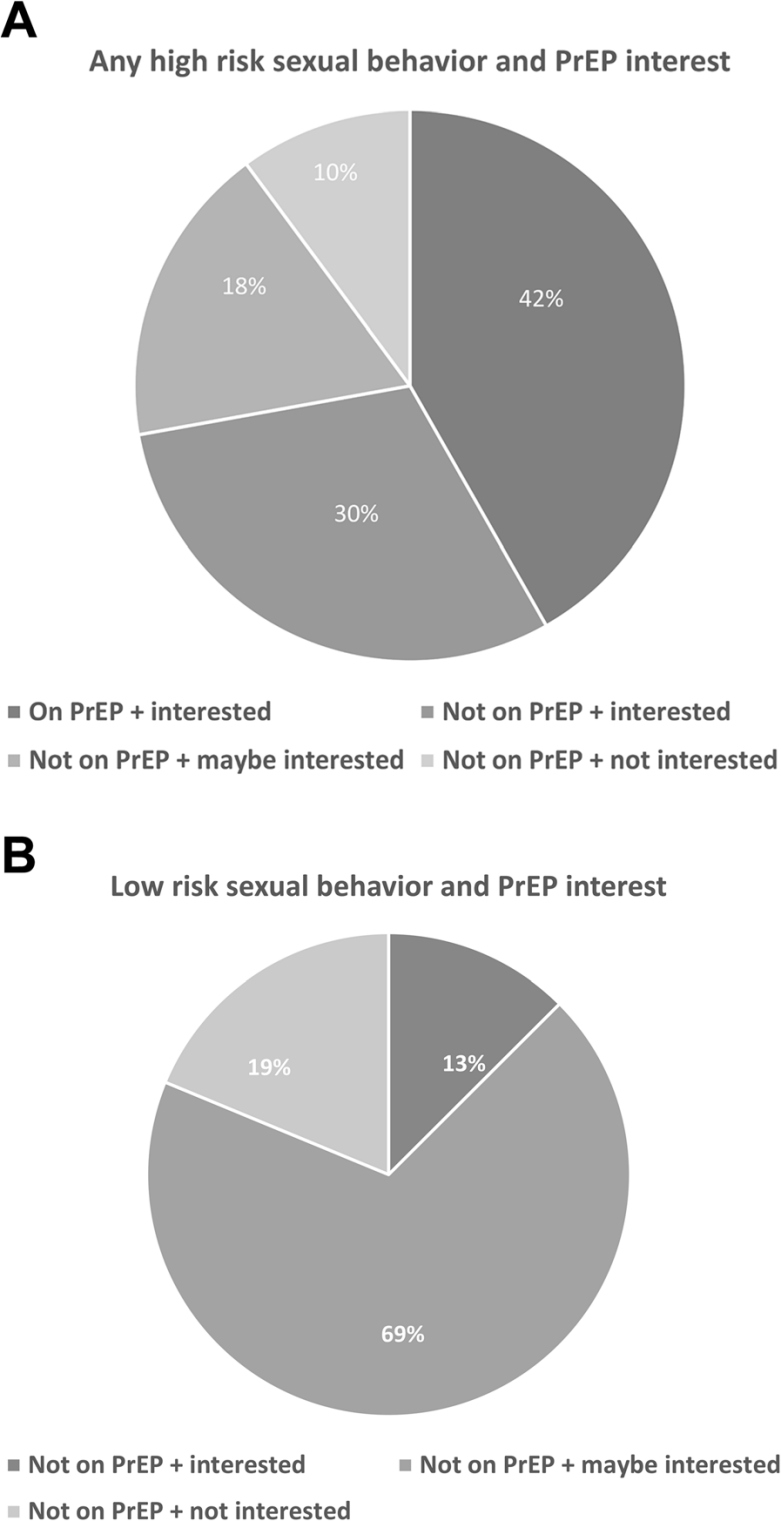
**a** High-risk behavior and PrEP interest, Fig. 1**b**. Low-risk behavior and PrEP interest *None of the individuals with low-risk behavior reported being on PrEP

Figure 1a presents a pie chart showing the proportion of participants who endorsed at least one high-risk behavior and their PrEP status: 42% were taking PrEP, 30% were interested in starting PrEP, 18% were not taking PrEP and needed more information, and 10% were not taking PrEP and not interested in PrEP. Fig. 1b presents a pie chart showing the proportion of participants who did not endorse at least one high-risk behavior and their PrEP status: none were taking PrEP, but 13% were interested in starting PrEP, 69% were not taking PrEP but needed more information, and 19% were not interested in PrEP. This difference was significant (*p* < 0.001).

### Acceptability of a peer navigator

Overall, 48% of participants were interested in a peer navigator for PrEP services, 41% were “maybe” interested, and 11% were not interested. Exploratory factor analysis confirmed that observed responses to questions on peer navigator acceptability reflected a single, unobserved latent variable or factor with an Eigenvalue of 3.33, associated with higher values of each response [36]. Factors with an Eigenvalue of 1 of greater are to be retained and are considered to be associated with the latent variable in question [37]. Uniqueness values ranged from 0.33 to 0.50; therefore, all question responses were retained. Rotated factor loadings were used to predict an index score for peer navigator acceptability for each participant, which was used as the primary outcome for regression analyses.

Race/ethnicity and sexual orientation were not independent predictors of peer navigator acceptability score, in unadjusted or adjusted analysis (Table 2). In the unadjusted model, having health insurance (beta = 0.55, 95% CI 0.11 to 0.99), higher sexual stigma score (beta = 0.05, 95% CI 0.01 to 0.09) and higher PHQ-9 score (beta = 0.04, 95% CI 0.01 to 0.07) were associated with higher peer navigator acceptability. Monthly income greater than $3500, relative to $0 to $1500 (beta = − 0.80, 95% CI − 1.26 to − 0.34) and having a regular medical provider (beta = − 0.63, 95% CI − 1.03 to − 0.23) were both associated with lower peer navigator acceptability. In the full multivariable model, no variables were associated with peer navigator acceptability. In the limited multivariable model, income greater than $3500 was negatively associated while sexual stigma was positively associated with peer navigator acceptability (adjusted beta = − 0.62, 95% CI − 1.10 to − 0.14; and adjusted beta = 0.04, 95% CI, 0 to 0.08, respectively).

**Table 2:** Regression analysis of factors associated with peer navigator acceptability

Attribute	Unadjusted beta (95% CI)	*P* value	Model 1 (full multivariable): Adjusted beta (95% CI)	*P* value	Model 2 (limited multivariable): Adjusted beta (95% CI)	*P* value
Sociodemographic factors						
Race/ethnicity						
Non-Latinx Black	Reference	0.17	Reference	0.83	0.15 (−0.30 to 0.61)	0.51
Latinx	0.30 (− 0.13 to 0.72)		−0.05 (− 0.57 to 0.47)			
Sexual orientation						
Gay	Reference		Reference		Reference	
Straight	− 0.98 (−2.30 to 0.34)	0.15	−1.09 (−2.72 to 0.24)		− 0.77 (−2.05 to 0.51)	0.32
Bisexual	0.04 (− 0.46 to 0.55)		− 0.02 (− 0.58 to 0.54)	0.26	0.07 (− 0.45 to 0.58)	
Queer	0.75 (− 0.11 to 1.60)		0.51 (− 0.41 to 1.44)		0.61 (−0.28 to 1.50)	
Age (years)	0.00 (−0.02 to 0.02)	0.98				
Education						
< High school	Reference					
High school	0.11 (−0.87 to 1.10)	0.30				
Some college	0.07 (−0.82 to 0.96)					
College	0.04 (−0.89 to 0.97)					
Graduate school	−0.67 (−1.71 to 0.38)					
Has health insurance	0.55 (0.11 to 0.99)	0.02	0.27 (−0.29 to 0.82)	0.34		
Monthly income ($)						
$0–$1500	Reference		Reference		Reference	
$1501–$3500	0.03 (−0.41 to 0.48)	0.001	0.06 (−0.41 to 0.53)	0.08	0.06 (−0.38 to 0.50)	0.02
> $3500	−0.80 (−1.26 to − 0.34)		−0.54 (− 1.06 to − 0.02)		−0.62 (− 1.10 to − 0.14)	
Foreign-born	0.34 (− 0.07 to 0.76)	0.10				
Current PrEP use and interest						
Currently taking PrEP (yes)	Reference					
Interested but not taking PrEP	0.77 (0.28 to 1.26)	0.005				
Not interested in taking PrEP	0.08 (−0.35 to 0.52)					
Mental health						
Stigma score	0.05 (0.01 to 0.09)	0.006	0.03 (−0.01 to 0.07)	0.14	0.04 (0 to 0.08)	0.03
PHQ-9 score	0.04 (0.01 to 0.07)	0.01	0.02 (−0.02 to 0.05)	0.36		
DAST score	−0.02 (− 0.10 to 0.06)	0.55				
AUDIT score	0.02 (−0.01 to 0.06)	0.17				
Healthcare factors						
Has regular medical provider	−0.63 (−1.03 to − 0.23)	0.002	− 0.29 (− 0.76 to 0.17)	0.21		
Disclosed same-sex behavior to provider	−0.31 (− 0.91 to 0.30)	0.32				
Sexual health						
Ever tested for HIV	0.37 (−0.37 to 1.10)	0.32				
Time since last HIV test						
< 3 months	Reference					
3–6 months	−0.06 (−0.57 to 0.46)	0.87				
6–12 months	−0.01 (− 0.70 to 0.69)					
> 12 months	0.25 (−0.41 to 0.91)					
Time since last clinic visit						
< 4 months	Reference					
4–12 months	−0.11 (−0.61 to 0.39)	0.89				
> 12 months	0.05 (−0.71 to 0.81)					
Reported condomless anal sex with a man in past 12 months	0.34 (−0.08 to 0.76)	0.11				
Currently in relationship with HIV-positive partner	0.20 (−0.54 to 0.94)	0.59				
Received STI treatment in past 12 months	0.27 (−0.13 to 0.67)	0.19				
Use of PEP in past 12 months	0.10 (−0.40 to 0.59)	0.70				
Any high-risk behavior	0.45 (−0.09 to 0.99)	0.10				
Use of crystal meth in past 12 months	0.51 (−0.14 to 1.20)	0.12				
Use of poppers in past 12 months	−0.20 (− 0.61 to 0.21)	0.34				
Non-prescription IVDU in past 12 months	0.54 (−0.42 to 1.50)	0.27				
Exchange sex for drugs, money, housing in past 12 months	0.45 (−0.34 to 1.24)	0.26				

*Missing some data for last HIV test

### Exploration of associations between sexual stigma and other variables

Because higher sexual stigma score was associated with higher peer navigator acceptability in these analyses, we used Wilcoxon rank sum tests to examine associations between sexual stigma score and the following dichotomous variables: ever having tested for HIV, report of any high-risk behavior, disclosure of MSM status to medical provider, report of any STD in the past 12 months, and current PrEP use. Having a STD in the past 12 months was the only variable tested that was significantly associated with higher sexual stigma score (*p* = 0.03; median stigma score 11.5 and 9 for those with and without an STD in the past 12 months, respectively). In addition, there were positive Spearman correlations between stigma score and both PHQ-9 score (*ρ* = 0.24, *p* = 0.02) and AUDIT score (*ρ* = 0.28, *p* = 0.01). No correlation was found between stigma and DAST scores (ρ = 0, *p* = 0.98).

### Peer attributes

**Fig. 2:**
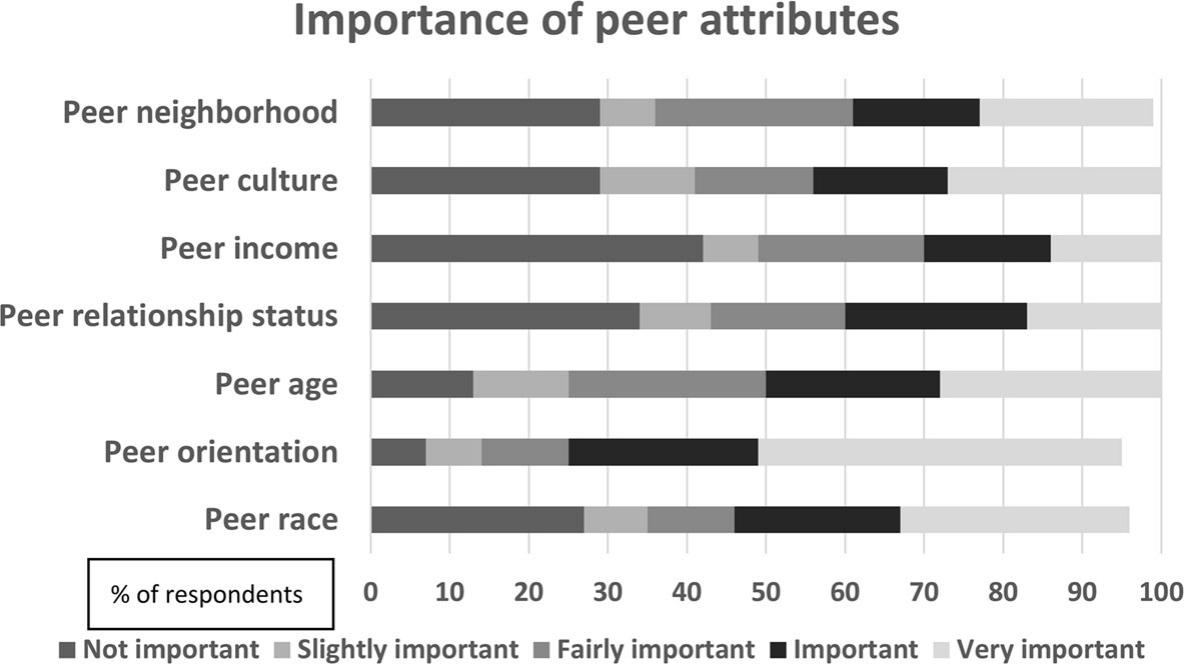
Likert scale responses for each peer attribute

**Fig. 3:**
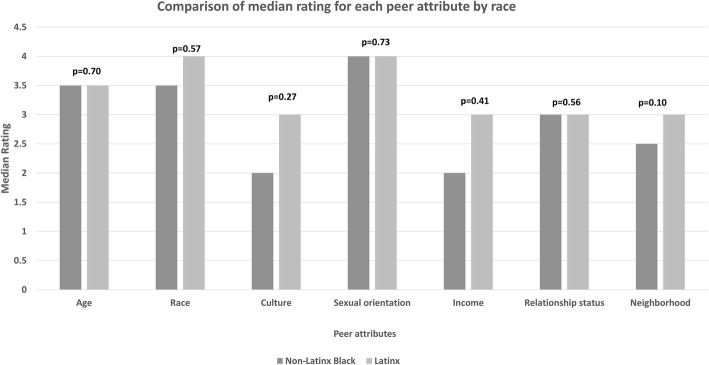
Comparison of median rating for each peer attribute by race/ethnicity Likert scale: (1) Not important (2) Slightly important (3) Fairly important (4) Important (5) Very important

Overall, 73% of participants rated having a peer navigator of the same sexual orientation as “important” or “very important.” The proportion of participants who rated sameness of peer attributes as “important” or “very important was 53% for race, 49% for age, 43% for culture, 38% for relationship status, and 38% for living in the same neighborhood (Fig. 2). The median rating by Latinx participants for same race, same culture, and same neighborhood were each higher when compared to the median rating by Black participants (Fig. 3), but these differences were not statistically significant. Spearman correlations between peer attribute rating and peer navigator acceptability were significant for several peer attributes: age (ρ = 0.29, *p* = 0.01), race (*ρ* = 0.27, *p* = 0.01), culture (*ρ* = 0.35, *p* = 0.0008), sexual orientation (*ρ* = 0.30, *p* = 0.01), and neighborhood (*ρ* = 0.34, *p* = 0.001) (Table 3).

**Table 3:** Correlation of peer attribute rating with peer navigator acceptability

Characteristic	Spearman correlation (ρ)	*P*-value
Same age	0.29	0.01
Same race	0.27	0.01
Same culture	0.35	0.0008
Same orientation	0.30	0.01
Same income	0.20	0.08
Same relationship status	0.20	0.07
Same neighborhood	0.34	0.001

## Discussion

The goal of our study was to gauge acceptability of a peer navigator for PrEP services among Black and Latinx MSM in western Washington. Overall interest in peer navigation was moderate, with just under half of participants expressing interest in PrEP navigation services, suggesting that peer navigation should be one of multiple strategies to engage MSM of color in HIV prevention efforts. Men with insurance and those with higher sexual stigma scores and higher PHQ-9 scores had higher acceptability scores, while men with higher income and regular medical providers had lower acceptability scores. Sexual stigma and income were independent predictors of peer navigator acceptability in multivariable analysis. Of interest, higher sexual stigma was associated with having a sexually transmitted infection (STI) in the past year. The majority of participants preferred a PrEP peer navigator with similar sexual orientation, race/ethnicity, and age as themselves. Overall, there were modest correlations between matching on peer attributes and peer navigator acceptability.

Our study did not require endorsement of sexual activity considered high risk by the CDC as an eligibility requirement, and we did not ask about number of sexual partners. There are multiple studies showing that minority MSM have small sexual networks compared to White MSM, with higher rates of HIV and bacterial STI acquisition despite this smaller size [38–40]. HIV acquisition is often heavily influenced by structural factors such as lack of insurance and access to healthcare, in addition to the usual risk factors for HIV seroconversion such as multiple sex partners, unprotected sexual encounters, and sex under the influence of drugs or alcohol [35, 41–43]. This rationale and a concern that sexual risk behavior is often underreported due to social desirability bias drove our selection of inclusion criteria for this study. In addition, we were interested in evaluating overall acceptance of peer navigation for PrEP among minority MSM as a group, as community norms are often an important factor in determining both stigma related to HIV prevention uptake and outcomes including the uptake of services [44], such as peer navigation for PrEP.

In the HIV Prevention Trials Network (HPTN) 061 study, which investigated strategies for HIV prevention among Black MSM in 6 major US cities, peer navigators were used to promote participant retention [45]. Participants who accepted peer navigation services were younger (*p* = 0.03) and more likely to be retained in the study than those who did not accept peer navigation (*p* < 0.001) [45]. At least one published study has found that gay identity was correlated with less social isolation and more willingness to engage with a peer outreach worker [46]. While sociodemographic factors (age, race/ethnicity, identification as gay) were not strong predictors of peer navigator acceptability among the Black and Latinx MSM in our study, these factors may still be important in other contexts and during actual PrEP delivery via peers.

In *What’s PrEP?,* higher monthly income was associated with lower peer navigator acceptability. Interestingly, the HPTN 073 study of PrEP uptake and adherence among Black MSM found that men with higher incomes had higher rates of PrEP adherence, suggesting that men with means may need limited or no help engaging with HIV prevention services [47]. In our study, having a regular medical provider was associated with lower peer navigator acceptability, although this factor was not significant in multivariable analysis. In a study by Santelli et al. among adolescents in the United States, confidential visits with a medical provider were associated with discussions about sensitive topics, including sexual orientation and HIV, suggesting that having a trusted regular provider increases access to HIV prevention services [48]. Similarly, although the association was not significant, peer navigator acceptability in our study was lower among participants who had disclosed their sexual orientation to a regular provider. Men who are “out” to their providers may feel that they do not need peer navigation services; conversely, a peer navigator may play an important supporting role when a medical provider is not LGBTQ-friendly. Finally, we found a positive association between having insurance and peer navigator acceptability, although this factor was not significant in multivariable analysis. One hypothesis to explain this finding is that individuals with insurance have fewer concerns about costs, and are therefore more willing to explore novel interventions for HIV prevention. In addition, having insurance is positively correlated with access to preventative health services, including PrEP [49].

Mental health has been identified as a barrier to HIV care engagement among young MSM, and peer navigation has been proposed to address this problem [50]. An ethnographic study of Black MSM in New York cited internalized homophobia, stigma, and family rejection as barriers to PrEP use, suggesting that men facing these issues might benefit from greater social support surrounding HIV prevention [51]. In the *What’s PrEP?* study, men with higher sexual stigma and depressive symptom scores were more accepting of a peer navigator, suggesting that minority MSM with greater emotional and psychiatric needs may be more open to PrEP peer navigation. Potentially, primary care providers could consider referring MSM with higher PHQ-9 scores for peer navigator services. However, depression was not a significant predictor of peer navigator acceptability in adjusted analysis, while sexual stigma, which is not usually assessed in clinical practice, was. Higher sexual stigma scores were also associated with an STI diagnosis in the past 12 months (*p* = 0.03). A recent STI diagnosis may present an important opportunity to reach out to at-risk MSM and offer peer navigation.

When asked about specific peer attributes, almost three quarters of participants ranked having a peer of the same sexual orientation as important or very important. Race, age, and culture were also ranked as important or very important by the majority of participants. Based on these findings, peer navigator programs to support PrEP use among MSM of color may need to match peers to clients on sexual orientation, race/ethnicity, and age group in order for programs to be effective. In our analysis, gender-queer or non-conforming participants were more accepting of peer navigator services for PrEP than gay or bisexual identified individuals, and straight-identifying individuals were less accepting, though these differences were not statistically significant. In prior studies involving peer navigation for homeless Black individuals, respondents indicated that having a peer with a similar lived experience was important to them [52]. Same-gender-loving individuals may feel more affirmed with a peer navigator of the same sexual orientation. There were modest positive correlations between specific peer attributes and peer navigator acceptability, with culture and neighborhood being the strongest. Matching on peer navigator attributes when possible may be helpful for programs using peer navigation to support PrEP uptake and adherence among minority MSM.

PrEP marketing that focuses on gay-identifying MSM may be stigmatizing to men in communities of color, especially those who do not identify as gay [53]. For such men, it may be important to emphasize PrEP’s value for sexual health and well-being, as opposed to focusing on risk [53, 54]. Our findings suggest that peer navigation for PrEP among Black and Latinx MSM may be more acceptable to men if they share a similar sexual orientation with the navigator – in many cases, this orientation may be bisexual or fluid. One potential strategy would be to triage minority MSM with a recent STI diagnosis for peer navigation matched on sexual orientation for discussions around STI prevention and information about PrEP.

Although the evidence base is still limited, several ongoing studies will examine the role of peer leaders or change agents in efforts to increase PrEP knowledge and use among MSM of color [55, 56]. In a cluster- randomized trial led by Patel et al. in New York City, an intervention focusing on “empowering with PrEP” (E-PrEP) using PrEP-focused social media messaging disseminated by a peer leader will be compared to an E-Health online control in which participants receive no PrEP-specific or HIV-related information [55]. In *PrEP Chicago*, the effectiveness of peer change agents recruited through social media networks and snowball sampling is being evaluated in an individual-level randomized trial evaluating outcomes including referrals to a PrEP informational hotline; PrEP knowledge, attitudes, and intention for use; and PrEP uptake among study participants [56]. Additional studies are needed to evaluate peer approaches in other settings, especially in areas such as western Washington in which minority communities are more dispersed and face significant barriers to accessing culturally tailored care. These studies should consider mechanisms through which peer support is thought to work, including the reduction of sexual stigma and the provision of social support from those of a similar background.

This study had several limitations. First, power was limited due to small sample size, particularly for Black participants, who only comprised about one third of our sample. In addition, we were unable to enroll any Black or Latinx transgender men. Although the *What’s PrEP*? study team included members of racial and sexual minorities and sought input and collaboration from community-based organizations serving MSM of color in the study area, effectively engaging this population was challenging for cisgender men and unsuccessful for transgender men of color. Mistrust of research may have been a factor, and specifically targeted research may be needed to reach vulnerable transgender men of color. This limited power decreased our ability to identify independent predictors of peer navigator acceptability in multivariable analysis. Second, the study took place in western Washington and our participants’ experiences may not be representative of those of MSM of color living in other parts of the United States. Third, online surveys are prone to fraudulent activity, and it is possible that there may have been duplicate surveys completed by the same individual using different contact information, despite the safeguards put in place. In addition, three survey entries for which contact details were not provided could not be confirmed as unique participants, and therefore were excluded. This may have resulted in underestimation or overestimation of peer navigator acceptability. Fourth, despite recruitment of both cis and transgender MSM using a two-step gender identity process, only cisgender MSM enrolled in our study. Perhaps partnering with a transgender-specific community-based organization would have helped to increase the participation of transgender MSM. Finally, because peer navigator acceptability in this study was measured in the abstract, actual acceptability remains unclear and may be higher or lower.

## Conclusion

Men of color who engage in HIV prevention efforts using PrEP may benefit from a peer navigator matched to their sexual orientation, race/ethnicity, age, and potentially other attributes. Men who may require additional emotional support or trauma-informed care due to stigma and discrimination, factors making them more vulnerable to HIV, may be more likely to seek out peer navigation in efforts to prevent HIV. MSM who had higher incomes were less accepting of a peer navigator, suggesting that offering peer navigation services to men with more means and greater access to care should not be a high priority. Clinics or organizations wishing to implement PrEP peer navigation programs for Black and Latinx MSM may want to consider the utility of matching peers to participants by sexual orientation, race/ethnicity, and age, as well as screening for sexual stigma and depression. Psychologically vulnerable MSM may have a greater need for a peer navigator, who may need to help men link to mental health services in addition to HIV prevention services. Furthermore, men with recent STI diagnoses and high sexual stigma scores may be appropriate targets for peer navigation to prevent HIV and other STI. Our results suggest that peer navigation is promising and should be incorporated into a suite of options for engagement and prevention of HIV among minority MSM, if it proves to be cost effective in future studies.

## Supplementary information

**Additional file 1.** What’s PrEP Study Questionnaire. Questions and response choices used in the study.

## Data Availability

The datasets analyzed during the current study are available in a REDCap database. They are not available publicly available due to the potential for individual privacy to be compromised, but a de-identified, limited dataset will be shared upon request to the corresponding author.

## Abbreviations

CI: Confidence interval
E-PrEP: Empowering pre-exposure prophylaxis
HIV: Human immunogenicity virus
HPTN: HIV prevention trials network
LGBTQ: Lesbian, gay, bisexual, transgender, queer
MSM: Men who have sex with men
PCAF: Pierce County AIDS Foundation
PHQ-9: Patient health questionnaire 9
PrEP: Pre-exposure prophylaxis
STD: Sexually transmitted disease
STI: Sexually transmitted infection
