# Supplementary material for: What’sPrEP?: peer navigator acceptability among minority MSM in Washington
Source: BMC Public Health. 2020 Feb 18;20:248. doi: 10.1186/s12889-020-8325-5 (PMC7029512; doi:10.1186/s12889-020-8325-5)
Supplement: Supplementary file 1 — Additional file 1. What’s PrEP Study Questionnaire. Questions and response choices used in the study. [file 12889_2020_8325_MOESM1_ESM.docx]

## Sociodemographic Questions

**Thank you very much for participating in this survey. Please answer some initial questions about who you are.**

1. Age in years: ___ ___
2. What is the highest level of education you have completed?
3. 12th grade or less
4. High school graduate or GED
5. Some college/AA degree/Technical school training
6. College graduate (BA or BS)
7. Graduate school degree: Master’s or Doctorate degree (MD, PhD, JD)
8. Relationship status
9. Single
10. Married/Partnered
11. Divorced/Separated
12. Other, specify: _____________
13. Occupational status
14. Employed full-time
15. Employed part-time
16. Full-time student
17. Casual/temp worker
18. Unemployed/not working due to disability
19. Retired
20. Were you born outside of the United States?
21. Yes
22. No

*[If NO ( b’) skip to Question 7. Otherwise, continue to Question 6]*

1. If yes, in what country were you born? __________________________________
2. Do you identify with any religious affiliation?

Yes

No

*[If NO ( b’) skip to Question 9. Otherwise, continue to Question 8]*

1. What religion do you identify with or practice?
   1. Catholic
   2. Protestant
   3. Muslim
   4. Hindu
   5. Other, specify: ________________________
2. Which of the following do you identify most closely with?
3. Gay, homosexual, or lesbian
4. Straight or heterosexual
5. Bisexual
6. Queer
7. Other, please specify: _______________________________
8. Don’t know

## Medical Care and Access Questions

**Next we have several questions about your access to community organizations and medical care.**

1. How were you recruited for this study?
2. On-line notification
3. E-mail from a friend or acquaintance
4. Community organization called POCAAN
5. Community organization called Neighborhood House
6. Community organization called Center for Multicultural Health
7. Community organization called Pierce County AIDS Foundation
8. Other, specify: ___________________________________
9. Do you currently have medical insurance?
10. Yes
11. No

*[If NO ( b’) skip to Question 13. Otherwise, continue to Question 12]*

1. What type of insurance do you have?
2. Private insurance through employer
3. Private insurance purchased through health exchange
4. Private insurance through my parents
5. Medicaid
6. Other, specify: _____________________________

*[Skip to question 15]*

1. Have you tried to get insurance?
2. Yes
3. No

*[If NO ( b’) skip to Question 15. Otherwise, continue to Question 14]*

1. What type of insurance have you tried to get?
   1. Private insurance through employer
   2. Private insurance purchased through health exchange
   3. Private insurance through my parents
   4. Medicaid
   5. Other, specify: _____________________________
2. How much money do you earn in a month before taxes are taken out? Please estimate in whole dollars: ___ ___ ___ ___
3. Do you have a regular healthcare provider?
   1. Yes
   2. No

*[If NO ( b’) skip to Question 18. Otherwise, continue to Question 17]*

1. Does this regular healthcare provider know you have sex with men?
   1. Yes
   2. No
2. How many months ago was your last clinic visit or medical professional contact?

Enter number: ___ ___

1. When you last visited a medical professional, where did you go?
   1. Private doctor/primary care provider
   2. Community clinic
   3. Public health clinic
   4. Hospital
   5. Emergency room
   6. Community-based organization
   7. Other, specify: __________________
2. Where do you get your health information?
   1. Regular health provider
   2. Other health service provider
   3. Online
   4. Friends
   5. Family
   6. Other, specify: _________________________

## HIV Prevention Attitudes and Knowledge

**HIV prevention now includes the use of anti-HIV (antiretroviral) medications, which can be taken after an exposure (post-exposure prophylaxis or PEP) or before an exposure (pre-exposure prophylaxis or PrEP) to protect against HIV. The next questions are about HIV testing, HIV prevention, and the use of PEP and PrEP.**

1. Have you ever tested for HIV before?
   1. Yes
   2. No

*[If NO ( b) skip to Question 24. Otherwise, continue to Question 22]*

1. When was your last HIV test? (Please give month and year): ___ ___ / ___ ___
2. Where did you get your last HIV test?
3. Private doctor/primary care provider
4. Community clinic
5. Public Health clinic
6. Hospital
7. Emergency room
8. Gay City
9. POCAAN
10. Neighborhood House
11. Center for MultiCultural Health
12. Pierce County AIDS Foundation
13. Planned Parenthood
14. Other community-based organization, specify: ______________________________________
15. Have you ever heard of HIV-negative people taking HIV drugs after a risky sexual exposure, in order to prevent HIV infection (this is called PEP, for Post-Exposure Prophylaxis)?
16. Yes
17. No
18. Don't remember

*[If NO ( b) or Don’t remember (c), skip to Question 26. Otherwise, continue to Question 25]*

1. Have you ever been given a prescription for PEP by a medical professional?
2. Yes
3. No
4. Have you ever heard of HIV-negative people taking HIV drugs every day to reduce their chances of getting HIV infection (this is called PrEP, for Pre-Exposure Prophylaxis)?
5. Yes
6. No
7. Don't remember

*[If NO (b) or Don’t remember (c), skip to Question 28. Otherwise, continue to Question 27]*

1. Have you ever been given a prescription for PrEP by a medical professional?
2. Yes
3. No
4. How confident are you to speak to a medical professional about your sexual health needs and concerns, including questions about PrEP?
5. Very Confident
6. Somewhat Confident
7. Somewhat Unsure
8. Very unsure
9. Don’t Know
10. Are you interested in starting or continuing PrEP?
11. Yes
12. No
13. Need more information
14. Don’t know/Unsure
15. Would you recommend PrEP to a friend or sexual partner?
16. Yes
17. No
18. Need more information
19. Don’t Know/Unsure
20. How do you feel about the following statement: “There should be more information about PrEP in my community.”
21. Strongly agree
22. Agree
23. Neutral
24. Disagree
25. Strongly disagree
26. Don’t Know/Unsure
27. How do you feel about the following statement: “There is a big need for PrEP in my community.”
    1. Strongly agree
    2. Agree
    3. Neutral
    4. Disagree
    5. Strongly disagree
    6. Don’t Know/Unsure
28. How do you feel about the following statement: “A lot of people in my community are taking PrEP.”
    1. Strongly agree
    2. Agree
    3. Neutral
    4. Disagree
    5. Strongly disagree
    6. Don’t Know/Unsure
29. How do you feel about the following statement: “My friends and family would support me if I told them I was taking PrEP.”
    1. Strongly agree
    2. Agree
    3. Neutral
    4. Disagree
    5. Strongly disagree
    6. Don’t Know/Unsure
30. Do you believe PrEP is an effective HIV prevention tool?
31. Yes
32. No
33. Need more information
34. Don’t Know/Unsure
35. What concerns do you have about taking PrEP? (Please check all that apply)
36. My risk is too low
37. I can’t afford it
38. I don’t think it works
39. I don’t know where to get PrEP
40. I don’t know enough about it
41. I hear there are side effects
42. I do not want to take a pill every day
43. I’m not a whore
44. I don’t like doctors
45. Don’t Know/Unsure
46. What would motivate you to start or continue PrEP? (Please check all that apply)
    1. If I knew enough about it
    2. If I felt that it worked
    3. If I felt I was at high risk for HIV
    4. If it was affordable
    5. If I knew where to get it
    6. If the side effects weren’t harsh
    7. If I was willing and able to take a pill every day
    8. If taking PrEP wasn’t stigmatized
47. If you wanted to get PrEP, where would you go?
    1. Private doctor/primary care provider
    2. Community clinic
    3. Public Health clinic
    4. Hospital-based clinic (such as Madison Clinic at Harborview)
    5. Gay City
    6. POCAAN
    7. Neighborhood House
    8. Center for MultiCultural Health
    9. Pierce County AIDS Foundation
    10. Planned Parenthood
    11. Other, specify: ________________________________
48. If you started or continued PrEP, where would you prefer to receive PrEP services?
49. Prefer location in my neighborhood
50. Would consider out-of-neighborhood location
51. Prefer out-of-neighborhood location

## HIV Risk Assessment Questions

**The next set of questions is about your risk for HIV infection, and focuses on behaviors that have been associated with an increased risk of HIV infection. Please answer truthfully; your responses are anonymous.**

1. Have you had anal sex without a condom outside of a mutually monogamous, long-term relationship with an HIV-negative man in the past 12 months?
   1. Yes
   2. No
2. Are you in an ongoing relationship with an HIV-positive male partner?
   1. Yes
   2. No
3. Have you been treated for an STD (syphilis, gonorrhea, chlamydia, etc) in the past 12 months?
4. Yes
5. No
6. Have you taken post-exposure prophylaxis (PEP, or antiretroviral drugs to prevent HIV) in the past 12 months?
   1. Yes
   2. No
7. Have you used crystal meth in the past 12 months?
8. Yes
9. No
10. Have you used poppers in the past 12 months?
11. Yes
12. No
13. Have you injected non-prescription drugs in the past 12 months?
14. Yes
15. No
16. Have you exchanged sex for drugs, money, or housing in the past 12 months?
17. Yes
18. No

## Sexual Stigma

### The next questions address how having male sex partners has affected your life and relationships.

1. How often have you heard that men who have sex with men are not normal?
   1. Never
   2. Once or twice
   3. A few times
   4. Many times
2. How often have you felt that you hurt or embarrassed your family because you have sex with men?
   1. Never
   2. Once or twice
   3. A few times
   4. Many times
3. How often have you been made fun of or called names because you have sex with men?
4. Never
5. Once or twice
6. A few times
7. Many times
8. How often have you been hit or beaten up because you have sex with men?
9. Never
10. Once or twice
11. A few times
12. Many times
13. How often have you had to pretend that you are not a MSM in order to be accepted?
14. Never
15. Once or twice
16. A few times
17. Many times
18. How often has your family not accepted you because you have sex with men?
19. Never
20. Once or twice
21. A few times
22. Many times
23. How often have you lost your friends because of you have sex with men?
24. Never
25. Once or twice
26. A few times
27. Many times
28. How often have you been kicked out of school for being a man who has sex with men?
29. Never
30. Once or twice
31. A few times
32. Many times
33. How often have you lost a place to live because you have sex with men?
34. Never
35. Once or twice
36. A few times
37. Many times
38. How often have you lost a job or career opportunity because you have sex with men?
39. Never
40. Once or twice
41. A few times
42. Many times
43. How often have you experienced police harassment because you have sex with men?
44. Never
45. Once or twice
46. A few times
47. Many times

###

### Mental Health

**Depression and anxiety are common conditions that can affect not only your mental wellbeing but also your physical health. The following questions are about your emotional health.**

1. Over the last 2 weeks, how often have you experienced little interest or pleasure in doing things?
   1. Not at all
   2. Several days
   3. More than half the days
   4. Nearly every day
2. Over the last 2 weeks, how often have you felt down, depressed, or hopeless?
   1. Not at all
   2. Several days
   3. More than half the days
   4. Nearly every day
3. Over the last 2 weeks, how often have you had trouble falling or staying asleep, or sleeping too much?
   1. Not at all
   2. Several days
   3. More than half the days
   4. Nearly every day
4. Over the last two weeks, how often have you felt tired or had little energy?
   1. Not at all
   2. Several days
   3. More than half the days
   4. Nearly every day
5. Over the last two weeks, how often have you had poor appetite or have overeaten?
   1. Not at all
   2. Several days
   3. More than half the days
   4. Nearly every day
6. Over the last two weeks, how often have you felt bad about yourself – or that you are a failure or have let yourself or your family down?
   1. Not at all
   2. Several days
   3. More than half the days
   4. Nearly every day
7. Over the last two weeks, how often have you had trouble concentrating on things, such as reading the newspaper or watching television?
   1. Not at all
   2. Several days
   3. More than half the days
   4. Nearly every day
8. Over the last two weeks, how often have you experienced moving or speaking so slowly that other people could have noticed? Or the opposite – being so fidgety or restless that you have been moving around a lot more than usual?
   1. Not at all
   2. Several days
   3. More than half the days
   4. Nearly every day
9. How difficult have any of the symptoms you experienced in the last 2 weeks made it for you to do your work, take care of things at home, or get along with other people?
   1. Not at all
   2. Somewhat difficult
   3. Very difficult
   4. Extremely difficult
   5. I had none of these symptoms in the last 2 weeks

## Substance Use

**Alcohol and other non-prescription drugs can also impact your mental wellbeing and physical health.**

**The following questions are about your use of alcoholic beverages.**

1. **How often do you have a drink containing alcohol?**
   1. Never
   2. Monthly or less
   3. 2 to 4 times a month
   4. 2 or 3 times a week
   5. 4 or more times a week

*[If response is Never (a), skip to question 76. Otherwise, continue to question 69.]*

1. How many alcoholic drinks do you have on a typical day when you are drinking?
2. 1 or 2
3. 3 or 4
4. 5 or 6
5. 7 to 9
6. 10 or more
7. How often do you have six or more drinks on one occasion?
8. Never
9. Less than monthly
10. Monthly
11. Weekly
12. Daily or almost daily
13. How often during the last year have you found that you were not able to stop drinking once you had started?
14. Never
15. Less than monthly
16. Monthly
17. Weekly
18. Daily or almost daily
19. How often during the last year have you failed to do what was normally expected of you because of drinking?
20. Never
21. Less than monthly
22. Monthly
23. Weekly
24. Daily or almost daily
25. How often during the last year have you needed a first drink in the morning to get yourself going after a heavy drinking session?
26. Never
27. Less than monthly
28. Monthly
29. Weekly
30. Daily or almost daily
31. How often during the last year have you had a feeling of guilt or remorse after drinking?
32. Never
33. Less than monthly
34. Monthly
35. Weekly
36. Daily or almost daily
37. How often during the last year have you been unable to remember what happened the night before because of your drinking?
38. Never
39. Less than monthly
40. Monthly
41. Weekly
42. Daily or almost daily

1. Have you or someone else ever been injured because of your drinking?
2. No
3. Yes, but not in the last year
4. Yes, during the last year
5. Has a relative, friend, doctor, or other health care worker ever been concerned about your drinking or suggested you cut down?
6. No
7. Yes, but not in the last year
8. Yes, during the last year

The following questions concern information about your use of drugs other than alcohol and tobacco during the past 12 months. These drugs include **marijuana, pain medications, sedatives, cocaine, amphetamines, crystal meth, heroin, and others.** Please remember that the questions do not refer to alcohol or tobacco use.

1. In the past year, have you used drugs other than those required for medical reasons?
   1. Yes
   2. No

*[If NO (b), skip to Question 89. Otherwise, continue to Question 79]*

1. In the past year, what type or types of non-medical drugs have you used (check all that apply)
2. Marijuana
3. Pain medications
4. Sedatives
5. Cocaine
6. Amphetamines
7. Crystal meth
8. Heroin
9. Other, please specify: ____________________
10. In the past year, have you abused more than one drug at a time?
11. Yes
12. No
13. In the past year, have you always been able to stop using drugs when you want to?
14. Yes
15. No
16. In the past year, have you had “blackouts” or “flashbacks” as a result of drug use?
17. Yes
18. No
19. In the past year, have you ever felt bad or guilty about your drug use?
20. Yes
21. No
22. In the past year, has your partner or your parent ever complained about your involvement with drugs?
23. Yes
24. No
25. In the past year, have you neglected your family because of your drug use?
26. Yes
27. No
28. In the past year, have you engaged in illegal activities in order to obtain drugs?
29. Yes
30. No
31. In the past year, have you ever experienced withdrawal symptoms (felt sick) when you stopped taking drugs?
32. Yes
33. No
34. In the past year, have you had medical problems as a result of your drug use (e.g. memory loss, hepatitis, convulsions, bleeding, etc…)?
35. Yes
36. No

## Support from a Trained Peer

**The research team is interested in finding out what might help minority MSM at risk for HIV infection, in terms of social support, help taking PrEP medications and keeping clinic appointments, and advice regarding “healthy living.” One intervention that may be helpful is the use of a peer who is similar to you in terms of background, has experience taking PrEP, and has received training on basic support and counseling. Trained peers would not be linked to any specific clinic, but instead would support men through the community, requesting support from clinic staff if and when needed. We call such individuals “peer navigators,” since they aim to help men navigate the health care system and other support services available.**

### Experience with Medication

1. Do you currently take any medication on a regular basis?
   1. Yes
   2. No

*[If NO ( b’) skip to Question 92. Otherwise, continue to Question 90.]*

1. What do you do to remind yourself to take your medicine?
   1. Nothing
   2. Alarm
   3. Phone reminder
   4. Mobile phone app
   5. Written system/calendar
   6. Other, describe: ________________________
2. How often do you miss pills in a month?
   1. Several times a week
   2. Once or twice a week
   3. Once or twice a month
   4. Only when I travel
   5. Never
3. Have you ever taken PrEP to prevent HIV infection?
   1. Yes
   2. No

*[If NO ( b’) skip to Question 95. Otherwise, continue to Question 93]*

1. Are you currently taking PrEP to prevent HIV infection?
   1. Yes
   2. No
2. Where did you get your PrEP medication? (Please check all that apply)
   1. Regular health provider
   2. STD clinic
   3. Gay City
   4. Kelley-Ross pharmacy
   5. A friend
   6. A sex partner
   7. Other, specify: ___________________________________________

### Peer Roles

1. If you were taking PrEP, how useful would it be to have someone other than clinic staff remind you of your clinic appointments and help you plan to be there on time?
   1. Very useful
   2. Somewhat useful
   3. Neither useful nor useless
   4. Somewhat useless or harmful/interfering
   5. Very harmful/interfering
2. How useful would it be to have someone assigned to help you by reminding you to take medication, especially in the first 1-2 months after starting PrEP?
   1. Very useful
   2. Somewhat useful
   3. Neither useful nor useless
   4. Somewhat useless or harmful/interfering
   5. Very harmful/interfering
3. Would you be willing to have a peer navigator (someone assigned to help you take your PrEP) get a notification by text message if you did not take your medication?
   1. Yes
   2. Maybe
   3. No
4. How useful would it be to have someone with experience taking PrEP who could provide advice on managing side effects, taking a daily pill, and planning for refills?
   1. Very useful
   2. Somewhat useful
   3. Neither useful nor useless
   4. Somewhat useless or harmful/interfering
   5. Very harmful/interfering
5. How useful would it be to discuss any concerns you have about privacy or other feelings about PrEP with another man of the same race/ethnicity as you who has experience taking PrEP?
   1. Very useful
   2. Somewhat useful
   3. Neither useful nor useless
   4. Somewhat useless or harmful/interfering
   5. Very harmful/interfering
6. Given these examples of ways a peer navigator could assist you, would you be willing to work with a peer navigator who would be assigned to you to help you prevent HIV infection by taking PrEP?
   1. Yes
   2. No
   3. Maybe
   4. Decline to answer

### Peer Attributes

1. How important would it be for a trained peer navigator to be the same racial/ethnic background as you?
   1. Very important
   2. Important
   3. Fairly important
   4. Slightly important
   5. Not at all important
2. How important would it be for a trained peer navigator to identify with the same sexual orientation as you (gay, straight, bisexual, etc.)?
   1. Very important
   2. Important
   3. Fairly important
   4. Slightly important
   5. Not at all important
3. How important would it be for a trained peer navigator to be around the same age as you?
   1. Very important
   2. Important
   3. Fairly important
   4. Slightly important
   5. Not at all important
4. How important would it be for a trained peer navigator to have the same relationship status as you (unattached, steady relationship, married, etc.)?
   1. Very important
   2. Important
   3. Fairly important
   4. Slightly important
   5. Not at all important
5. How important would it be for a trained peer navigator to have an income similar to yours?
   1. Very important
   2. Important
   3. Fairly important
   4. Slightly important
   5. Not at all important
6. How important would it be for a trained peer navigator to be from the same culture (Afro-Caribbean, Hispanic, Ethiopian, etc.) as you?
   1. Very Important
   2. Important
   3. Fairly Important
   4. Slightly Important
   5. Not Important
7. How important would it be for a trained peer navigator to be from the same Seattle area neighborhood as you?
   1. Very Important
   2. Important
   3. Fairly Important
   4. Slightly Important
   5. Not Important
8. How would you prefer to communicate with a trained peer navigator? (check all that apply)
   1. In person
   2. By telephone
   3. By e-mail
   4. By text messaging
   5. Other, please specify: _________________________________
9. How often would you be willing to communicate with a trained peer navigator?
   1. Daily
   2. A few times a week
   3. Once a week
   4. Once or twice a month
   5. Less than once a month

### Additional Interventions

1. How helpful would daily or weekly text messages or phone calls be for helping you remember to take your pills?
   1. Extremely
   2. Very
   3. Moderately
   4. Slightly
   5. Not at all
2. How helpful would text messages or phone call reminders from your peer navigator be for helping you attend your clinic appointments?
   1. Extremely
   2. Very
   3. Moderately
   4. Slightly
   5. Not at all
3. How concerned would you be about privacy issues due to text messages or phone calls about taking your medications or attending clinic appointments?
   1. Extremely
   2. Very
   3. Moderately
   4. Slightly
   5. Not at all
4. How useful do you think you would find an app for your cell phone to remind you to take your medicine and to send you (or your peer navigator) a notification that you have not yet taken your pill that day?
   1. Extremely
   2. Very
   3. Moderately
   4. Slightly
   5. Not at all
5. How useful would a pill organizer that could hold and organize one week or one month of your medications be to ensure you can take your pills every day?
   1. Extremely
   2. Very
   3. Moderately
   4. Slightly
   5. Not at all
6. How helpful do you think it would be if your provider spent 5-10 minutes at each appointment making sure that all of your concerns had been heard and addressed (to the extent possible)?
   1. Extremely
   2. Very
   3. Moderately
   4. Slightly
   5. Not at all
7. How useful would you find monthly counseling to problem-solve and help motivate you to take your pills every day?
   1. Extremely
   2. Very
   3. Moderately
   4. Slightly
   5. Not at all
8. Motivational interviewing is a method where care providers work with patients to identify barriers to medication adherence (e.g. not wanting to take pills at home due to non-disclosure) and to find ways for the patient to self-motivate to take their pills. If your care provider suggested motivational interviewing would you be willing to try it?
   1. Yes
   2. No

## Conclusion

**Thank you very much for taking the time to answer these questions. All responses are completely confidential and will not be linked to any identifying information.**

**Please feel free to contact the organization that referred you for this survey if you would like assistance with any of the issues discussed in this study, including getting health insurance, access to PrEP, or referrals for mental health or substance abuse counseling.**
